# The role of tau in neurodegeneration

**DOI:** 10.1186/1750-1326-4-13

**Published:** 2009-03-11

**Authors:** Tania F Gendron, Leonard Petrucelli

**Affiliations:** 1 Department of Neuroscience, Mayo Clinic College of Medicine, Jacksonville, Florida, USA

## Abstract

Since the identification of tau as the main component of neurofibrillary tangles in Alzheimer's disease and related tauopathies, and the discovery that mutations in the tau gene cause frontotemporal dementia, much effort has been directed towards determining how the aggregation of tau into fibrillar inclusions causes neuronal death. As evidence emerges that tau-mediated neuronal death can occur even in the absence of tangle formation, a growing number of studies are focusing on understanding how abnormalities in tau (e.g. aberrant phosphorylation, glycosylation or truncation) confer toxicity. Though data obtained from experimental models of tauopathies strongly support the involvement of pathologically modified tau and tau aggregates in neurodegeneration, the exact neurotoxic species remain unclear, as do the mechanism(s) by which they cause neuronal death. Nonetheless, it is believed that tau-mediated neurodegeneration is likely to result from a combination of toxic gains of function as well as from the loss of normal tau function. To truly appreciate the detrimental consequences of aberrant tau function, a better understanding of all functions carried out by tau, including but not limited to the role of tau in microtubule assembly and stabilization, is required. This review will summarize what is currently known regarding the involvement of tau in the initiation and development of neurodegeneration in tauopathies, and will also highlight some of the remaining questions in need of further investigation.

## Introduction

The accumulation of proteinaceous aggregates is a pathological hallmark of many neurological diseases characterized by neuronal dysfunction and eventual cell death. In tauopathies, as the name aptly implies, these aggregates take the form of neurofibrillary tangles (NFT) composed of tau. This group of diseases includes Alzheimer's disease (AD), frontal temporal dementia with Parkinsonism linked to chromosome 17 (FTDP-17), progressive supranuclear palsy, Pick's disease and corticobasal degeneration. In contrast to AD, for which the deposition of NFT occurs only in neurons, tau-positive inclusions are observed in glial cells in a variety of tauopathies [1]. Each tauopathy exhibits a characteristic regional pattern of NFT formation and the degeneration of vulnerable neuronal networks follows a stereotypical pattern. For example, NFT are distributed primarily to the entorhinal region, hippocampus and cortex in AD, to the brain stem, basal ganglia and cerebellum in progressive supranuclear palsy and to the frontal and temporal cortex in FTDP-17. Despite their diverse phenotype and distinct clinical presentations, common to all tauopathies is the progressive accumulation of NFT composed of insoluble, hyperphosphorylated tau in a filamentous form, such as twisted or straight filaments or paired helical filaments (PHF).

Tau was first isolated in 1975 as a protein that co-purifies with tubulin and has the ability to promote microtubule assembly *in vitro *[2,3]. As one of the principal components of the cytoskeletal system, microtubules are involved in the maintenance of neuronal morphology and the formation of axonal and dendritic processes. In addition to structural support, microtubules play a vital role in cellular trafficking. By providing tracts for motor proteins, like kinesins and dynein, they enable the transport of cargo to specific parts of the cell. The cargo transported to and from pre- and postsynaptic sites is critical for synaptic function and includes mitochondria, components of synaptic vesicles and plasma membranes, ion channels, receptors and scaffolding proteins. Synapses are highly vulnerable to impairments in transport; therefore perturbations in this system could cause malfunctions in neurotransmission and signal propagation and lead to synaptic degeneration.

The polymerization, stability and organization of microtubules are regulated by microtubule-associated proteins, such as MAP1, MAP2 and tau. Tau predominantly localizes to neuronal axons where it modulates the stability and assembly of microtubules. In so doing, tau generates a partially stable, but still dynamic, state in microtubules important for axonal growth and effective axonal transport. Tau, in a distinct phosphorylated form, is also present in the somatodendritic compartment of neurons, as well as in astrocytes and perineuronal glial cells [4,5]. In addition to binding microtubules, some studies [6-11], but not all [12], provide evidence that tau can interact, either directly or indirectly, with actin and affect actin polymerization as well as the interaction of actin filaments with microtubules. Tau may also interact with the plasma membrane [13-15] and with several proteins involved in signal transduction [16-22].

**Figure 1:**
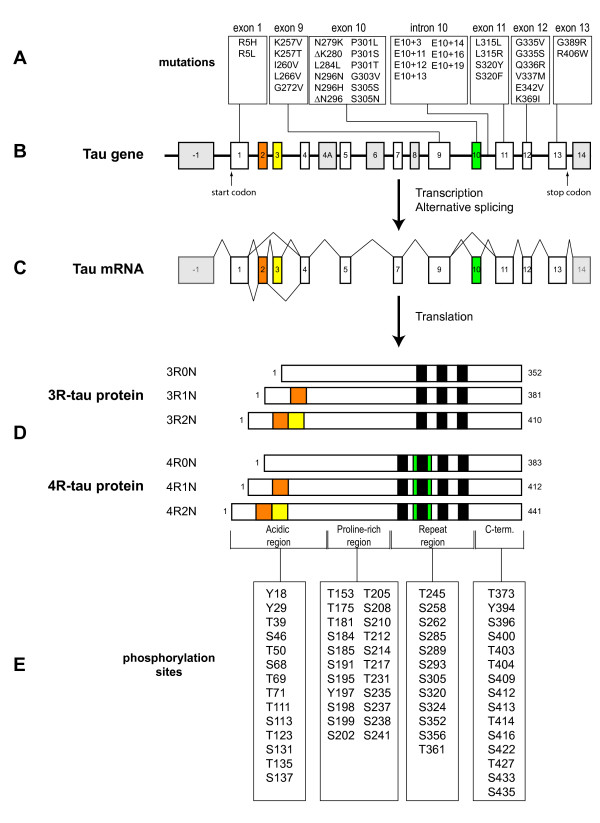
**A schematic representation of the human tau gene, mRNA and protein isoforms**. The human tau gene is located on chromosome 17q21 and contains 16 exons (panel B). White boxes represent constitutive exons and the gray or colored boxes represent alternatively spliced exons. Identified mutations in exons 1–13, and intron 10, of the tau gene are shown using the numbering of the 441-amino acid isoform of tau (panel A). Exon -1 is part of the promoter and is transcribed but not translated, as is the case for exon 14 (panel C). Exons 4A, 6 and 8 are not transcribed in human. Exons 2, 3 and 10 are alternatively spliced, as demonstrated by the different lines linking these exons (panel C), generating a total of 6 different mRNAs which are translated into six different tau isoforms (panel D). These isoforms differ by the absence or presence of one or two N-terminal inserts encoded by exon 2 (orange box) and 3 (yellow box), as well as the presence of either three or four repeat regions coded by exons 9, 10, 11 and 12 (black boxes) in the C-terminus. The second repeat, encoded by exon 10, is highlighted in green. Panel E indicates sites in the acidic, proline-rich, repeat and C-terminal regions of tau reported to be phosphorylated *in vivo *or *in vitro*.

The tau protein is encoded by the *MAPT *gene located in chromosome 17 [23]. In the adult human brain, alternative mRNA splicing of exons 2, 3 and 10 yields six tau isoforms (Fig. 1). The isoforms differ by the absence or presence of one or two acidic inserts at the N-terminal, and whether they contain three or four repeats of a conserved tubulin binding motif at the C-terminal [24]. The repeat region, present within the microtubule binding domain, binds to microtubules and promotes their assembly. Tau isoforms with four repeats (4R-tau) bind microtubules with a greater affinity than isoforms with three repeats (3R-tau), and can even displace the previously bound 3R-tau [25]. Phosphorylation of certain residues within the repeat region impairs the interaction between tau and microtubules, leading to the detachment of tau [26]. The N-terminal half of tau, known as the projection domain because it protrudes from the surface of microtubules, includes the acidic region and a proline-rich region. The projection domain is proposed to determine the spacing between microtubules [27], and may play a role in the interactions between tau and other cytoskeleton proteins, like neurofilament proteins [28]. Additionally, this domain associates with the plasma membrane [13,14] and the PPXXP or PXXP motifs in the proline-rich region are important for the association of tau with certain proteins containing Src homology 3 domains (SH3).

Though the gene encoding tau is not genetically linked to AD, mutations in *MAPT *cause FTDP-17 [29,30], and missense mutations have also been found in progressive supranuclear palsy [31], corticobasal degeneration [32,33] and in conditions that closely resemble Pick's disease [34,35], thus providing evidence that disrupting tau homeostasis suffices to cause neurodegeneration (Fig. 1). Tau gene polymorphisms have also been described and two different haplotypes, H1 and H2, have been identified [36]. The H1 haplotype is a risk factor for progressive supranuclear palsy and corticobasal degeneration, perhaps due to increased tau expression or the imbalanced expression of alternative tau transcripts (for review, see [37]).

Tau mutations are known to alter the relative proportion of various tau isoforms [29], impair the ability of tau to bind and promote the assembly of microtubules [38-40], or enhance the aggregation of tau into filaments [41,42]. It is therefore expected that tau-mediated neurodegeneration is caused by a combination of toxic gains of function incurred by abnormalities in tau, as well as from the harmful consequences that result from the loss of normal tau functions. Unfortunately, the exact mechanisms by which abnormalities in tau initiate, or contribute, to neuronal demise are not entirely understood. This review will summarize what is currently known regarding the role of tau in the initiation and development of neurodegeneration in AD and related tauopathies, and will also highlight some of the remaining questions in need of further investigation.

### Filaments and neurotoxicity

NFT are one of the most striking pathological features in tauopathies; therefore, much attention has focused on understanding how the deposition of NFT cause neurodegeneration, in essence using a top-down approach to investigating the mechanism of disease. Though the tau hypothesis of neurodegeneration is evolving, it has long been postulated that the aggregation of tau into filaments and NFT results in a toxic gain of function. In AD, the number of NFT in the neocortex positively correlates with the severity of cognitive decline [43], and several missense mutations in tau that cause frontotemporal dementia accelerate tau filament assembly *in vitro *[42,41,44]. Thus, it is assumed that NFT are directly able to induce neuronal damage. Yet, given that tau is normally a highly soluble protein that does not readily aggregate into filaments, this matter has been difficult to assess in experimental models because of the resistance of tau to aggregate within an ideal time-frame for culture studies or within an animal's relatively short lifespan. Further complicating matters is evidence that mouse tau appears to prevent tau aggregation in transgenic mice overexpressing wild-type human tau (htau) [45]. By crossing tau knockout (tau-/-) mice with transgenic 8c mice that express all six isoforms of htau, Andorfer et al., (2003) generated mice that exclusively express htau (called htau mice) [45]. These htau mice develop AD-like pathology, with hyperphosphorylated tau accumulating as aggregated PHF. Conversely, even though 8c mice express high levels of both htau and mouse tau, they do not develop tau pathology. Normal adult mouse brains contain only 4R-tau isoforms, so the main difference between 8c and htau mice is the presence of mouse 4R-tau. It is thus likely that mouse 4R-tau protects transgenic mice expressing non-mutant htau from developing neurofibrillary pathology. Nonetheless, transgenic mice that overexpress high levels of htau isoforms containing aggregation-promoting mutations (e.g. P301L tau) can develop tau pathology even in the presence of endogenous mouse tau [46-48]. To accelerate tau aggregation *in vitro*, polyanionic cofactors or small molecule ligands are often used to facilitate tau fibrilization. For example, in a cell culture model overexpressing full-length tau, Congo red treatment stimulates the formation of filamentous tau aggregates and decreases cell viability [49]. Since tau overexpression is not toxic in the absence of the aggregation-inducer, these results suggest that tau aggregation causes cell death or, at least, accelerates its onset.

Because high concentrations of tau are required to promote tau fibrilization in experimental models, it is believed that the enhanced ability of tau to form filamentous inclusions in the cytoplasm of neurons and glia in human tauopathies may be due to pathological conditions that increase the pool of tau available for aggregation. Elevated levels of free tau, not bound to microtubules, would presumably enhance the assembly of tau into oligomers and could increase its likelihood to become misfolded, as well as undergo modifications or conformational changes that promote the formation of insoluble filamentous inclusions. Yet, while tau protein levels are increased in the AD brain [50], it is unlikely that the amount of tau in various tauopathies is as high as in cell culture and animal models that artificially force tau overexpression. It remains possible, however, that local tau concentrations may be increased in restricted areas of the cell during disease and this initiates the polymerization of tau leading to NFT formation.

There are a number of ways by which NFT may damage neurons and glial cells. For example, by acting as physical barriers in the cytoplasm, NFT would compromise normal cellular functions. In transgenic mice expressing mutant (P301L) htau, the accumulation of tau filaments in the cell body of neurons not only displaces many cytoplasmic organelles from their usual location but also decreases the number of normal organelles [51]. Of interest, PHF-tau, either isolated from AD brains or generated *in vitro*, inhibits proteasome activity [52], and could therefore unfavorably perturb cellular homeostasis. In a similar fashion, the proteasome activity in HEK293 cells stably expressing tau is decreased following tau hyperphosphorylation and aggregation [53]. These findings are consistent with the notion that protein aggregates are not inert end-products but actively influence cell metabolism, like proteasomal activity [54]. NFT may also cause neuronal toxicity by reducing normal tau function. Since tau is redistributed to filaments in AD [55], and since filamentous tau does not promote microtubule assembly *in vitro *[56], the sequestration of tau to NFT may disrupt tau-mediated regulation of microtubule dynamics. However, the reduction in microtubule number and length observed in AD does not correlate with the presence of PHF [57,58]. Furthermore, whether the loss of functional tau alone is sufficient to cause microtubule destabilization is under some debate. Tau deficiency does delay the maturation and extension of neurites in embryonic neuronal cultures [59,60] but no major cytoskeletal abnormalities are observed in adult tau-/- mice [61]. The lack of an obvious phenotype in tau-/- mice is most probably due to a redundancy in function among tau and other microtubule-associated proteins and their apparent compensation for the loss of tau [62]. Nevertheless, the overt breakdown of the microtubule system may not be required to cause neuronal injury. For instance, several mutations that cause tau dysfunction and neuronal death in FTDP-17 alter the ratio of 4R-tau to 3R-tau, and these isoforms differentially modulate microtubule dynamics [63]. Thus, less obvious changes in the regulation of microtubules may have harmful consequences. In any event, NFT need not alter microtubule integrity in order to aberrantly affect one of the main functions of microtubules, namely fast axonal transport. In a recent study, LaPointe et al., (2008) demonstrated that filaments formed by the longest isoform of htau impair anterograde, but not retrograde, transport in isolated squid axoplasm without producing changes in microtubule morphology [64]. Filaments of htau appear to inhibit anterograde transport by dissociating kinesin-1 from its vesicular cargo and this effect is mediated by protein phosphatase 1 (PP1) and glycogen synthase kinase-3 (GSK-3) [64]. Overall, NFT may cause toxicity by a number of mechanisms though questions remain as to whether NFT are the main culprit of tau-induced toxicity.

### Non-filamentous tau intermediates and neurotoxicity

The development of NFT is initiated by the formation of pre-tangles of oligomeric tau that assemble into insoluble filaments before aggregating to form NFT. Prior to, during or after this process, tau undergoes numerous, and potentially harmful, modifications. Therefore, though NFT may themselves be neurotoxic, the presence of some of these modifications may be indicative of tau-mediated damage that arose before their deposition. Indeed, tau-mediated neuronal death, in the absence of tau filaments, is observed in Drosophila and some transgenic mouse models overexpressing htau [65-67]. Mice overexpressing htau with the P301L mutation (rTg4510 mice) do develop age-related NFT, neuronal loss and memory impairments. Yet, the subsequent suppression of the mutant tau stabilizes neuronal loss and improves memory function even though NFT continue to accumulate [47]. In rTg4510, a regional dissociation between neuronal loss and the accumulation of NFT is observed; there is a loss of neurons in the dentate gyrus before NFT lesions appear and, conversely, NFT appear without major cell loss in the striatum [68]. Likewise, many of the neurons that accumulate NFT in aged transgenic mice overexpressing normal htau seem "healthy" in terms of nuclear morphology, while a number of dying neurons do not appear to have a significant load of tau filaments [69]. Furthermore, using models based on quantitative data on neuron loss and NFT formation as a function of disease duration, it is estimated that CA1 hippocampal neurons in AD can survive with NFT for approximately 20 years [70]. Together, these studies suggest that tau-mediated neuronal death does not require the formation of NFT. Rather, non-filamentous tau, as well as abnormally modified tau intermediates, may be neurotoxic. Indeed, tau can undergo numerous post-translational modifications and some of these modifications, like phosphorylation and glycosylation, are believed to occur early in the development of tau pathology [71,72]. However, it is not yet known which tau intermediates are critical for the development of the different stages of neurodegeneration and by which mechanisms these intermediates cause cellular injury.

#### Tau hyperphosphorylation

The phosphorylation of tau plays a physiological role in regulating the affinity of tau for microtubules. Though less well studied, phosphorylation also regulates the binding of tau to signaling molecules and could thus influence tau-mediated signaling [21]. Most of the phosphorylation sites on tau are present in the proline-rich and the C-terminal regions flanking the microtubule binding domains (Fig 1); (for review, see [73]). The kinases that phosphorylate tau can be divided into two major groups, according to motif specificity: proline-directed protein kinases (PDPK) and non-proline-directed protein kinases (non-PDPK). The PDPK include cyclin-dependent kinase 5 (cdk5), mitogen-activated protein kinase, and several stress-activated protein kinases. GSK3-β is often described as a PDPK but the proline is not always required for phosphorylation by GSK3-β. Both cdk5 and GSK3-β co-purify with microtubules [74,75] and phosphorylate tau within a cellular environment [76,77]. The phosphorylation of tau by these kinases inhibits the ability of tau to promote microtubule assembly and facilitates the polymerization of tau into PHF [78-81]. Among the non-PDPK are cyclic AMP-dependent protein kinase (PKA), calcium- and calmodulin-dependent protein kinase II (CaMKII), and microtubule affinity regulating kinase (MARK), the mammalian homologue of PAR-1. MARK targets KXGS motifs within the microtubule binding repeat domains (serine residues at 262, 293, 324 and 356) of tau [82]. Tau phosphorylation at KXGS motifs induces its dissociation from microtubules and prevents its degradation [83]. Unbound tau may then be hyperphosphorylated by other kinases. In fact, the phosphorylation of tau by MARK/PAR-1 may be a prerequisite for the action of downstream kinases, including GSK-3β and Cdk5 [84]. There is also evidence that tau can be phosphorylated on tyrosine residues (Tyr18, Tyr29, Tyr197 and Tyr394) [85-89].

Tau hyperphosphorylation is an early event in the pathogenesis of tauopathies, appearing before the development of NFT [71]. Several missense mutations (G272V, P301L, V337M and R406W) in FTDP-17 result in tau proteins that are more favorable substrates to kinases *in vitro *[90]. In AD brains, the levels of total tau are approximately eight-fold higher than in age-matched controls, and this increase is due to higher levels of abnormally hyperphosphorylated tau, either polymerized into NFT of PHF or straight filaments, or present as a non-fibrillized form in the cytosol [50,91]. Elevated levels of hyperphosphorylated tau are also detected in cerebral spinal fluid of AD patients and may be predictive of neurodegeneration [92,93]. The increase in tau protein is not likely to result from increased transcription since several studies failed to observe increased tau mRNA levels in AD brains compared to controls [94-98], though one study did report a relative downregulation of 3R-tau mRNA and an upregulation of 4R-tau mRNA in areas heavily affected by NFT [99]. Since these studies did not examine tau mRNA expression at the cellular level, it remains possible that differences in tau mRNA levels between AD and normal cases occur in selective cell subpopulations. Interestingly, while one study found no change in tau mRNA isoform expression in AD, it did find that levels of mRNA for 4R-tau isoforms were increased in the brainstem, but not the fontal cortex or cerebellum, of patients with progressive supranuclear palsy [98].

There is ample experimental evidence to support the view that hyperphosphorylated tau plays a pathological role in tauopathies. For example, the expression of pseudophosphorylated tau, which mimics disease-like tau hyperphosphorylation, causes apoptosis in neuronal cells, an effect not observed when cells express wild-type tau [100]. The co-transfection of tau with GSK-3β in a cell culture model results in more cell death compared to the expression of tau and mutant (inactive) GSK-3β, suggesting that tau phosphorylation by GSK3-β is toxic [101]. In a similar fashion, the activation of cdk5 by overexpressing p25 accelerates tau phosphorylation and aggregation in mice overexpressing mutant (P301L) tau [102]. In fact, p25 overexpression and the ensuing cdk5 activation even contribute to tau pathology in mice expression only endogenous tau. Some studies have shown that p25 transgenic mice show increased tau phosphorylation compared to wild-type controls and, although NFT are not present, cytoskeletal components are disorganized, axonal swelling is observed, and the affected axoplasm is filled with abnormally clustered mitochondria and lysosomes, features consistent with loss of a functional microtubule network [103,104]. Cruz et al., (2003) also examined cdk5 activation on tau pathology and this group used bitransgenic mice that inducibly overexpress human p25 in the forebrains of mice. In these mice, a time-dependent increase in neuronal loss and astrogliosis is observed in the cerebral cortex between 5 and 12 weeks of cdk5 induction. Tau phosphorylation is increased in p25 transgenic mice compared to controls but there is no marked change in total tau protein levels. By 27 weeks of cdk5 induction, NFT pathology is visible in the cerebral cortex and hippocampus [105]. Together, these results provide compelling evidence that aberrant tau hyperphosphorylation can lead to neurodegeneration, even in the absence of tau mutations or forced tau overexpression. Of interest, cdk5 activity is elevated in the prefrontal cortex of AD brains, where NFT are found, but not in the cerebellar cortex suggesting a relationship between deregulated cdk5 activity and tau pathology in humans [106,107].

Not only may increased kinase activity participate in tau hyperphosphorylation, but so may decreased tau dephosphorylation. Tau is dephosphorylated by protein phosphatase 2A (PP2A) and, to a lesser extend, by PP1, PP2B and PP5 [19,108-110]. In the human brain, PP2A, PP1, PP5 and PP2B account for approximately 71, 11, 10 and 7%, respectively, of the total tau phosphatase activity [110]. The mRNA and protein expression of some phosphatases, as well as their activities, are decreased in affected areas of AD brain [96,110-114]. For example, in the AD hippocampus, PP2A and PP1 mRNA levels are decreased [111] and the protein expression level of PP2A subunits is significantly and selectively decreased in AD-affected brain regions and in tangle-bearing neurons [114]. Indeed, the progressive loss of PP2A subunit expression closely parallels the formation of tau lesions in discrete neurons [114]. Compared to controls, phosphatase activity towards hyperphosphorylated tau is lower in gray matter extracts from AD brains [112] and PP2A activity is decreased in homogenates from the frontal and temporal cortices [114]. Of interest, one study found that the activities of PP2A and PP5 are decreased in the AD brain but PP2B activity is increased [110]. Nonetheless, the total phosphatase activity in this study was significantly lower [110] and another study has shown PP2B activity to be decreased in the AD brain [113]. Together, these findings suggest that the downregulation of phosphatase activity, especially that of PP2A, can contribute to increasing levels of hyperphosphorylated tau. Consistent with this notion, PP2A inhibition by okadaic acid induces tau hyperphosphorylation and accumulation in rat brain slices [109] and the inhibition of PP2A and PP1 activity by calyculin A injections into the rat hippocampus leads to tau hyperphosphorylation and defects in spatial memory retention [115]. Moreover, transgenic mice with reduced neuronal PP2A activity exhibit increased tau hyperphosphorylation and the accumulation of tau aggregates in the soma and dendrites of cortical pyramidal cells and cerebellar Purkinje cells [116].

Tau phosphorylation is also regulated by Pin1 (protein interacting with NIMA 1), a member of the peptidyl-prolyl *cis-trans *isomerase group of proteins involved in the assembly, folding and transport of cellular proteins. The interaction between tau and Pin1 depends on the phosphorylation state of tau; Pin1 binds tau when phosphorylated at Thr231 [117] and facilitates its dephosphorylation by PP2A [118-120]. In AD neurons, Pin1 binds hyperphosphorylated tau in PHF, potentially depleting soluble Pin1 levels [117,121]. Pin1 is significantly down-regulated and oxidized in the AD hippocampus [122]. Additionally, pyramidal neurons from AD brains that have lower Pin1 levels are more prone to contain tangles, whereas neurons with higher levels of Pin1 are generally tangle-free [123]. Deregulation of Pin1 expression and activity could induce an imbalance in the phosphorylation-dephosphorylation of tau and negatively impact tau regulation and function. Indeed, Pin1 restores the ability of phosphorylated tau to bind microtubules and promote microtubule assembly *in vitro *[117]. It has been proposed that Pin1 functions as a co-chaperone and, together with HSP90 and other members of the HSP90 complex, is involved in the refolding and dephosphorylation of aberrantly phosphorylated tau [83]. If Pin1 levels are knocked-down in Hela cells by siRNA prior to transfecting cells with wild-type tau, tau levels are decreased compared to Pin1-expressing cells [83]. This suggests that when Pin1 levels are decreased, attempts to refold/dephosphorylate tau are subverted and tau degradation is favored. However, Pin1 knock-down *increases *the stability of wild-type tau, as well as that of V337M and R406W mutant tau in SH-SY5Y cells [124]. Differences in the results among these two studies may reflect differences in the culture models used and experimental design. It is also possible that, in the absence of Pin1 and its associated dephosphorylation and refolding activities, the degradation machinery may become overburdened, leading to tau accumulation. It should also be noted that, while knocking-down Pin1 increases the stability of wild-type tau and various mutant forms of tau in SH-SY5Y cells, it decreases the stability of P301L- and P301S-tau [124] indicating that the effect of Pin1 on tau is mutation-dependent. Of interest, Pin1-/- mice develop age-dependent neuropathy, characterized pathologically by tau hyperphosphorylation, tau filament formation and neuronal degeneration in the brain and spinal cord [123], thus providing another model in which the hyperphosphorylation of endogenous tau correlates with neuronal death. Conversely, Pin1 overexpression reduces tau levels and suppresses the tauopathy phenotype in transgenic mice expressing wild-type tau [124]. However, in keeping with the opposing effects of Pin1 on wild-type tau and P301L-tau in SH-SY5Y cells, Pin1 overexpression exacerbates the tauopathy phenotype in P301L tau transgenic mice. Moreover, when Pin1-/- mice are crossed with transgenic mice overexpressing mutant (P301L) tau, P301L mutant tau levels are decreased and the robust tauopathy phenotype is abolished [124].

Though many questions remain regarding the cause of aberrant tau phosphorylation in tauopathies, tau hyperphosphorylation is believed to play an important role in tau-mediated toxicity. Soluble hyperphosphorylated tau isolated from AD brains has lower microtubule-promoting activity *in vitro *[125] and sequesters normal tau, MAP1 (A/B) and MAP2, causing the inhibition of microtubule assembly and even the disassembly of microtubules [126,127]. These findings suggest that hyperphosphorylated tau can cause the breakdown of microtubules by interacting with microtubule associated proteins. Consequently, one could thus speculate that hyperphosphorylated tau is involved in the depletion and abnormal orientation of microtubules that is observed in the frontal cortex layers II and III in AD brains [58]. An expected consequence of disarrayed or depleted microtubules is the impairment of microtubule-based transport, also an early event observed in AD [128,129]. As previously mentioned, loss of tau function alone may be insufficient to disrupt microtubule networks [61]. However, the combined loss of tau and other microtubule-associated proteins could have more detrimental consequences on microtubule regulation. Consistent with this is the observation that mating tau-/- and MAP1B-/- mice leads to a lethal postnatal phenotype [62].

Unlike the soluble form of hyperphosphorylated tau, the filamentous form of tau does not bind MAPs and does not disrupt microtubules *in vitro *[56]. Not only does this imply that tau filaments would have less of an impact on the microtubule network, the formation of filaments may, in fact, be a mechanisms adopted by neurons to sequester the toxic forms of hyperphosphorylated tau. However, if NFT *are *detrimental to cells, and if tau hyperphosphorylation facilitates aggregation and filament formation, this could be one more mechanism by which tau hyperphosphorylation contributes to neuronal death. When hyperphosphorylated tau isolated from the AD brain is dephosphorylated by PP2A, the ability of tau to polymerize into PHF is inhibited. Conversely, the sequential rephosphorylation of tau by PKA, CaMKII, and GSK3-β or cdk5, as well as by GSK3-β and cdk5, promotes the assembly of tau into tangles of PHF similar to those observed in the AD brain [130]. Yet, the *in vitro *phosphorylation of recombinant tau promotes the formation of tau filaments in some studies [130,131] but not all [132], putting into question the role of tau phosphorylation in enhanced filament formation.

Another mechanism by which tau hyperphosphorylation may contribute to neuronal toxicity is through its interaction with actin. In *Drosophila *and mice, tau leads to the accumulation of filamentous actin into structures resembling the Hirano bodies observed in the brains of patients with AD or other tauopathies, like Pick's disease [11]. Hirano bodies are intraneuronal inclusions that contain, among other proteins, actin and tau [133,134], and may play a causative role in AD [135,136]. The formation of Hirano body-like structures in neurons disrupts microtubules in neurites and could thus impair axonal transport and lead to synapse loss [135]. Fulga et al., (2007) have shown that phosphorylated tau can induce changes in the actin cytoskeleton and lead to toxicity. The retinal expression of pseudophosphorylated tau in *Drosophila *induces a striking accumulation of actin in the lamina and produces substantial toxicity. Conversely, the expression of phosphorylation-incompetent tau does not lead to actin accumulation and only causes mild toxicity [11]. These results suggest that phosphorylated tau can cause neuronal death by inducing changes in the actin cytoskeleton.

Overall, though tau hyperphosphorylation is implicated in tau pathology, it is still not fully understood which of the tau phosphorylation sites are critical for the development of tauopathies, nor is it decidedly known how hyperphosphorylated tau causes neuronal death. A better understanding of the physiological roles of tau phosphorylation, as it regulates the binding of tau to microtubules and affects other less well characterized functions of tau, will likely shed light on the mechanisms by which tau hyperphosphorylation contributes to cell death.

#### Other tau modifications

Intimately linked to tau phosphorylation is tau glycosylation. Glycosylation is characterized by the covalent attachment of oligosaccharides to protein side chains. Glycosidic bonds are classified as either N-linked or O-linked. In N-linked glycosylation, the sugar is linked to the amide group of asparagine residues of proteins, while in O-linked glycosylation, sugars are attached to a hydroxyl group of serine or threonine residues. Hyperphosphorylated tau and PHF-tau purified from AD brains are glycosylated, mainly through N-linkage [137,138]. Additionally, non-hyperphosphorylated tau isolated from AD brains is also glycosylated, whereas no glycan is detected in tau purified from normal control brains [137], suggesting that aberrant glycosylation precedes abnormal tau hyperphosphorylation. Indeed, glycosylation facilitates the site-specific phosphorylation of tau catalyzed by PKA, cdk5 and GSK-3β [137,139]. Conversely, glycosylation appears to inhibit the dephosphorylation of tau by PP2A and PP5 [140]. Tau glycosylation may also coordinate with hyperphosphorylation to stabilize the filamentous structure of PHF given that deglycosylation of PHF untwists PHF into straight filaments [137]. Together, these findings suggest that aberrant N-linked glycosylation is an early tau modification that enhances tau hyperphosphorylation, which may drive NFT formation, and also help maintain and stabilize NFT structures.

In addition to N-linked glycosylation, human brain tau can be modified by O-linked monosaccharide β-N-acetylglucosamine (O-GlcNAc) [141]. O-GlcNAcylation regulates tau phosphorylation in a site-specific manner in both cultured cells overexpressing htau and in rodent brains; at most of the phosphorylation sites examined, O-GlcNAcylation reduces tau phosphorylation [141]. Consistent with this finding, in neuroblastoma cells transfected with htau, O-GlcNAc mainly modifies the less-phosphorylated tau species, while highly phosphorylated tau is devoid of O-GlcNAc residues [142]. In starved mice, a model used to mimic the reduction in glucose uptake and metabolism observed in the AD brain, O-GlcNAcylation is decreased and tau hyperphosphorylation is increased in the brains of the mice [141]. In the AD brain, the level of O-GlcNAcylation is lower than that in control brains, indicating that O-GlcNAcylation is compromised [141]. Based on these findings, it was proposed that impaired glucose metabolism in AD may contribute to disease pathogenesis by reducing tau O-GlcNAcylation and, consequently, increasing tau phosphorylation [143]. Yuzwa et al., (2008) have shown that Thiamet-G, an inhibitor of O-GlcNAcase that enhances O-GlcNAcylation, markedly reduces tau phosphorylation in PC12 cells at pathologically relevant sites, like Thr231 and Ser396. Moreover, Thiamet-G also efficiently reduces phosphorylation of tau at Thr231, Ser396 and Ser422 in both the rat cortex and hippocampus [144]. Together, these findings underscore the dynamic relationship between the O-GlcNAcylation and phosphorylation of tau.

Besides phosphorylation and glycosylation, tau undergoes other changes that could enhance tau self-assembly and filament formation and may confer toxic gains or loss of function. For instance, the proteolytic cleavage of tau coincides with the pathogenesis of AD. Granular aggregations containing tau truncated at Glu391 are detected within the somatodendritic compartment of AD brains but not in age-matched non-demented controls [145], Glu391-truncated tau is present in PHF isolated from AD tissue [146-148] and tau-truncated at Asp421 associates with neurofibrillary pathology in AD brains [149-151]. Tau cleaved at Glu391 and/or Asp421 is also observed in Pick's disease, progressive supranuclear palsy and corticobasal degeneration [152-154].

The truncation of tau accelerates its assembly into fibrils *in vitro *[149,155,156], promotes microtubule assembly *in vitro *more than full-length tau [157], and increases its association with microtubules [158]. The effect of tau phosphorylation at Ser396/Ser404 on microtubule binding differs between full-length tau and tau truncated at Asp421, indicating that specific tau forms (e.g. intact versus cleaved tau) respond differently to site-specific phosphorylation [158]. Notably, transgenic rats that overexpress truncated tau species (aa 151–391) in the brain and spinal cord develop neurofibrillary pathology [157], and cultured cortical neurons derived from these rats have fewer mitochondria in neuronal processes, display higher levels of reactive oxygen species and are more susceptible to oxidative stress compared to cultures from non-transgenic rats [159]. Consistent with these findings, the expression of tau fragments cause cell death or render cells more sensitive to insults in various culture models [160-163].

Taken together, the above findings suggest that tau cleavage is neurotoxic. However, there is some debate as to whether tau cleavage occurs before or after the aggregation of tau into NFT. On the one hand, Guillozet-Bongaarts et al., (2004) have shown by immunohistochemical studies that tau truncation at Asp421 occurs only after the Alz50 conformation change in tau, the presence of which is indicative of the appearance of filamentous tau [164]. On the other hand, the deletion of CHIP, a tau ubiquitin ligase, leads to the accumulation of non-aggregated, hyperphosphorylated and caspase-cleaved tau in mice, suggesting that tau hyperphosphorylation and caspase-3 cleavage both occur prior to aggregate formation [165]. Indeed, Rissman et al. (2004), show that in both transgenic mice and in AD brain, caspase-cleaved tau at Asp421 associates with early and late markers of NFT and correlates with cognitive decline [150].

In addition to the incorporation of truncated tau into NFT, the PHF and NFT in AD brains are glycated [166] as well as ubiquitinated [167,168], but these modifications are believed to be later events in disease progression. Nitrated tau is also detected in cytoplasmic inclusions in AD, corticobasal degeneration, Pick's disease, progressive supranuclear palsy and FTPD-17 [169]. Tau-nY29, an antibody specific for tau when nitrated at Tyr29, detects soluble tau and PHF-tau from severely affected AD brains but fails to recognize tau from normal aged brains, suggesting that tau nitration is disease-specific [170]. The exact mechanisms by which nitrated tau contributes to pathology, however, remain poorly understood. Nitration can greatly affect protein folding and function [171,172]. Peroxynitrite (ONOO-), which is capable of both protein nitration and oxidation [173], leads to tau oligomerization *in vitro *and in neuroblastoma cells [174,175]. Yet, it is believed that this effect results from the oxidative role of peroxynitrite and the formation of dityrosine bonds in tau [175]. The overall effect of tau nitration by peroxynitrite *in vitro *is to delay the polymerization of tau into filaments [175,176]. The toxicity of tau nitration may instead result from the inhibitory effect of nitration on the ability of tau to promote tubulin assembly which could compromise microtubule function [177].

#### Tau mutations

Even though no mutations in tau have been identified in AD or sporadic cases of frontotemporal dementia, understanding how mutations in tau confer toxicity in FTDP-17 should provide insight on the role of tau in the development of neurodegeneration. At least 34 mutations in the human *MAPT *gene, falling into two functional classes, have been reported (Fig. 1) [178]. The first class of mutations, which includes missense and deletion changes in the coding region of *MAPT*, generates tau proteins with altered function. These mutations can reduce the binding affinity of tau for microtubules [38,39]. LeBoeuf et al., (2008) have shown that FTDP-17 tau mutations that map to the repeat/inter-repeat region of tau compromise its ability to regulated microtubule dynamics *in vitro *[179]. However, cells transiently expressing mutant (P301L or R406W) or wild-type tau are indistinguishable in terms of the co-localization of tau with microtubules and the generation of microtubule bundles [180], implying that these tau mutations do not have an immediate impact on the integrity of the microtubule system. In addition to impaired microtubule binding, first class mutations enhance the ability of tau to aggregate and form filaments *in vitro *[41,42,44]. Insoluble aggregates in patients with the P301L mutation consist largely of mutant 4R-tau, with only small amounts of normal 4R- and 3R-tau [181]. The selective trapping of P301L tau in the insoluble deposits is presumably caused by the increased aggregation potential conferred by the mutation. It is tempting to speculate that the combined effects of altered microtubule regulation and accelerated NFT formation caused by mutations in tau contribute to tau-mediated toxicity or, at the very least, render cells more vulnerable to age-related stressors.

The second class of mutations affects the alternative splicing of *MAPT *transcripts, mainly influencing exon 10 splicing and leading to a change in the ratio of tau isoforms with three of four microtubule binding repeats. In the normal adult brain, the ratio of 4R- to 3R-tau is approximately 1. Many of the second class mutations increase this ratio [29], suggesting that 4R-tau is the more toxic isoform. However, while only 4R-tau aggregates into twisted and straight filaments in corticobasal degeneration and progressive supranuclear palsy, NFT in AD brains contain both 3R- and 4R-tau, and 3R-tau inclusions are primarily observed in Pick's disease [182-184]. Therefore, neurodegeneration may not result from one isoform being more toxic than another, but rather from an imbalance in the proper ratio of 3R- to 4R-tau. One hypothesis proposes that since splicing mutations cause an excess of a specific tau isoform and, since 3R- and 4R-tau bind microtubules at different sites [185], a shortage of available binding sites would occur for the overexpressed tau isoform [186]. This could lead to an excess of free tau available for filament assembly. It is also highly probable that abnormal changes in isoform expression would adversely affect tau function. Given that various tau isoforms are differentially expressed during development, differentially distributed in neuronal subpopulations and even present in distinct localizations within neurons [187], it is likely that they have specific functions. For example, different tau isoforms have dramatically different effects on the rate and number of motors driving the cargo along microtubules [188]. As our understanding of the functions carried out by distinct tau isoforms grows, so will our understanding of how alterations in their expression levels contribute to neuronal dysfunction.

### Mechanisms of tau toxicity

#### Impaired axonal transport and synaptic damage

It is clear that tau undergoes several abnormal modifications during the evolution of tauopathies. Different tau intermediates are likely to play various roles in the onset and progression of disease and several modifications of tau may have converging mechanisms of toxicity. While many questions remain, a better understanding of the early events in tau-mediated toxicity is especially important as it may lead to the development of therapeutic strategies that prevent the pathological events that initiate neuronal dysfunction. Synaptic damage is an early event in AD [189] and synapse loss correlates with cognitive deficits even more strongly than the number of NFT [190,191]. In addition to AD, synapse loss is reported in other tauopathies, like progressive supranuclear palsy [192] and frontal lobe degeneration of non-Alzheimer type [193,194].

Animal models of tauopathy provide evidence that defects in tau can cause synaptic damage. Yoshiyama et al. (2007), show that hippocampal synaptic loss is observed in transgenic mice overexpressing P301S htau (PS19 mice) before NFT formation [195]. These mice develop early synaptic pathology; a prominent decrease in levels of the pre-synaptic proteins, synaptophysin and β-synuclein, is detected in the CA3 region of hippocampus by 3 months of age. To examine the functional consequence of synaptic pathology, *in vivo *electrophysiology was conducted using 6 month old PS19 mice, an age that precedes marked NFT formation and neuronal loss. At this age, synaptic conduction, presynaptic function and long-term potentiation, thought to underlie learning and memory, are impaired in PS19 mice compared to non-transgenic controls. In agreement with this study, Eckermann et al., (2007) reported that a reduction in the number of spine synapses in tau transgenic mice occurs in the absence of NFT formation. For their study, two transgenic mouse lines were created. One line expresses full-length htau with the ΔK280 mutation that strongly promotes tau aggregation. The second line contains the same ΔK280 mutation and two additional proline mutations (ΔK280/PP) to disrupt aggregation. The hyperphosphorylation of tau and the missorting of tau to the somatodendritic compartment are observed in both mutants but conformational changes in tau are observed only in the pro-aggregation mice. Of particular interest, though the formation of NFT is not observed in either line, synapse loss is greater in the transgenic animals expressing the pro-aggregation mutant of tau compared to animals expressing the anti-aggregation mutant. This suggests that the ability of tau to form oligomers is likely to hasten synaptic decline while supporting the notion that overt filament formation is not necessary for synaptic loss [196]. In agreement with this, the accumulation of early-stage aggregated tau species, which antecedes the formation of NFT, is associated with the development of functional deficits during the pathogenic progression in rTg4510 mice [197]. As observed in mice, tau-induced synaptic dysfunction is seen before any evidence of neuronal death or NFT formation in *Drosophila *[198]. The overexpression of htau in larval motor neurons causes a disruption of axonal transport and reduces the number of detectable mitochondria in the presynaptic terminals of neuromuscular junctions. Tau-expressing neuromuscular junctions are functionally abnormal, exhibiting disrupted vesicle cycling and impaired synaptic transmission.

Various mechanisms by which non-fibrillar tau could disrupt axonal transport and cause synaptic damage have been proposed. One possibility is that tau hyperphosphorylation leads to microtubule disassembly and loss of the tracks needed for transport. As previously mentioned, soluble hyperphosphorylated tau isolated from AD brains has decreased microtubule-promoting activity *in vitro *[125,199] and sequesters normal tau, MAP1 (A/B) and MAP2, causing the inhibition of microtubule assembly and even the disassembly of microtubules [126,127]. In so doing, the hyperphosphorylation of tau may destabilize microtubules, thus impairing the microtubule tracks needed for the transport of molecular motors and their cargo. A second possibility is that transport inhibition results from too much tau binding microtubules and essentially blocking the movement of motor proteins [200,201]. The transfection of htau in mature hippocampal neurons results in the overexpression and improper distribution of tau so that it invades dendrites in addition to axons. The high levels of tau cause transport inhibition of mitochondria. This may be because tau either displaces motor proteins from microtubules or prevents their association to microtubules by covering the microtubule surface. Additionally, tau overexpression causes microtubules to bundle and this further impedes mitochondrial movement, leading to mitochondrial degeneration, loss of ATP and synaptic degeneration [201]. In this model, tau-mediated synaptic loss is delayed by overexpressing the kinase MARK2/PAR-1, which increases tau phosphorylation at the KXGS motif. Since phosphorylation of tau at this site detaches tau from microtubules, it is thought that MARK2/PAR-1 activation postpones synaptic degeneration by removing tau from the microtubule tracks and reversing the transport block. It should be kept in mind that, although modifications in tau may lead to its accumulation in tauopathies, the overexpression of tau in this model may increase tau levels beyond what is observed in disease. Finally, evidence is now emerging that the ability of tau to impair axonal transport does not necessarily involve microtubule dysfunction. As it happens, tau itself binds kinesins [202,203] and is transported along axons as kinesin cargo [204]. This raises the possibility that high levels of unbound tau may compete with potential kinesin cargo and thus prevent their translocation to the synapse. Indeed, co-immunoprecipitation experiments show that when full-length tau is overexpressed in differentiated NB2a/d1 cells, the binding of kinesin to vimentin and neurofilament medium (NF-M) is decreased, presumably because these proteins are displaced from kinesin by tau [202]. Furthermore, when tau is co-transfected in cells overexpressing NF-M, the anterograde transport of NF-M is selectively decreased while the percentage of non-moving NF-M, as well as NF-M exhibiting retrograde transport, increases [202]. Since retrograde transport is not impaired, it is unlikely that the inhibition of anterograde axonal transport resulting from tau overexpression is caused by altered microtubule dynamics. In contrast to these findings, the perfusion of full-length htau, at a physiological concentration, does not reduce anterograde fast axonal transport in isolated squid axoplasm [64]. However, when axoplasm is perfused with tau isoforms lacking the C-terminus, anterograde (but not retrograde) transport is inhibited [64]. Together, these results suggest that tau modifications or its accumulation beyond normal physiological levels, are required for tau to affect axonal transport. Notably, Cuchillo-Ibanez et al., (2008) report that the phosphorylation state of tau regulates its ability to bind kinesin-1; tau phosphorylated by GSK-3 associates with the light chain of kinesin-1 more than dephosphorylated tau [203]. Of interest, in cortical neurons transfected with full-length tau, the inhibition of GSK-3 reduces tau phosphorylation and decreases the rate of fast axonal transport of tau. In contrast, tau pseudophosphorylation mutants for GSK-3 sites are transported significantly faster compared to wild-type tau [203]. Based on the above findings, it is tempting to speculate that hyperphosphorylated tau would be better than normal tau at scavenging kinesin and displacing other kinesin cargo, thus preventing their anterograde axonal transport.

It ought to be mentioned that, although filament deposition may not be necessary for tau-mediated transport inhibition and synapse loss, it is likely to enhance synaptic damage. In lamprey central neurons that overexpress the shortest isoform of htau, tau filament formation appears to precede the beading of distal dendrites and the progressive loss of dendritic microtubules and synapses [205]. In this model, filament assembly occurs surprisingly rapidly; neurons expressing htau for 5–10 days contain densely packed htau filaments throughout their somata and dendrites. In this system, synaptic loss may be caused by large NFT that physically obstruct the movement of mitochondria along microtubules or may be due to the ability of NFT to inhibit fast axonal transport by triggering the release of cargo from kinesin [64].

#### Aberrant tau-mediated intracellular signaling

Though the role of tau in regulating microtubule dynamics is well established, much less is known regarding the role of tau in other cellular functions. Given the ability of tau to interact with the plasma membrane and to bind a variety of proteins, tau is proposed to participate in cell signaling. Potential signaling proteins that bind tau include PP1 [18], PP2A [19], the scaffolding protein 14-3-3 [20] and phospholipase Cγ (PLCγ1) [16,21]. Additionally, tyrosine kinases (Fyn, cSrc, Lck and Fgr), the p85a regulatory subunit of phosphatidylionositol 3-kinase and PLCγ1 have been shown to bind tau through their SH3 domains [17,21]. SH3 domains recognize the PXXP motif in proteins, seven of which are present in htau close to known tau phosphorylation sites. The binding of tau to signaling molecules implies that tau is either a substrate to the binding enzyme or that tau regulates the activity of the protein to which it is bound. With some binding partners, both situations may be true. For example, tau is not only phosphorylated by Fyn [85,206] it also modulates Fyn activity [207]. Tau increases PLCγ activity *in vitro *[208], and also increases Fyn and Src kinase activity both in *in vitro *assays and within COS7 cells [207]. Additionally, tau primes Src for activation in 3T3 cells stimulated with platelet-derived growth factor, as reflected by sustained actin stress fiber breakdown [207]. These results suggest that tau can impact actin remodeling by upregulating Src tyrosine kinase activity.

It is worth noting that the phosphorylation of tau alters its ability to bind SH3 domains [21,206]. Tau isolated from normal human brain is able to bind SH3 domains but PHF-tau isolated from AD brains cannot [21]. Similarly, the interaction between tau and the plasma membrane is modulated by the phosphorylation state of tau [14,15,209]. In human neuroblastoma cells [209] and in PC12 cells [15], plasma membrane-bound tau is less phosphorylated than cytoplasmic or total tau. Furthermore, when PC12 cells are transfected with wild-type htau, a substantial amount of tau is isolated in the plasma membrane fraction. In contrast, when cells are transfected with tau pseudophosphorylation mutants to mimic PHF-tau, no tau is present in the plasma membrane fraction [15]. Thus, abnormal alterations in the phosphorylation state of tau may aberrantly affect its association with the plasma membrane and with various signaling proteins. It is not yet known if other abnormal tau modification would do so as well.

#### Tau-enhanced vulnerability

Several forms of neurotoxicity are hypothesized to be involved in the etiology of AD. Among them are inflammation, oxidative stress, mitochondrial dysfunction, calcium dysregulation and excitotoxicity. Though none of these are specific to AD, as they occur in a variety of neurodegenerative diseases and/or with ageing, abnormalities in tau may accelerate their development or render neurons more vulnerable to these insults. For instance, tau-mediated disruption of intracellular transport, and especially defects in mitochondria trafficking and the ensuing decrease in ATP levels, may not only impair normal neurotransmission, but may also render neurons more susceptible to age-related stressors. For example, mitochondrial dysfunction increases the susceptibility of neurons to excitotoxicity, the pathological process by which excessive activation of glutamate receptors leads to neurodegeneration [210,211]. Also, mitochondrial dysfunction can provoke the release of presynaptic glutamate and impair the clearance of glutamate from the synapse, thus leading to high levels of extracellular glutamate and sustained glutamate receptor activation [212-214]. In fact, cell death from tau overexpression in cultured neurons is dependent on the activation of NMDA receptors, a subtype of glutamate receptor [215]. While not yet studied, NMDA receptor activation by tau overexpression may be due to increased glutamate levels caused by altered mitochondria trafficking or by a decrease in the expression of glutamate transporters. Mice overexpressing tau in astrocytes show decreased expression and function of the glial glutamate transporter, GLT-1 [216]. In addition to potentially provoking excitoxic insults, Roberson et al., (2007) propose a role for tau in modulating sensitivity to such insults. The intraperitoneal injection of kainate, a glutamate receptor agonist, dose-dependently induces seizures in tau+/+ mice. In contrast, tau+/- and tau-/- mice are resistant to kainate-induced seizures [217]. In a similar fashion, compared to tau+/+ mice, tau+/- and tau-/- mice are protected against the behavioral deficits caused by overexpressing the human amyloid precursor protein [217]. Tau reduction also provides protection against β-amyloid toxicity in primary neurons [218,219]. For example, cultured hippocampal neurons obtained from wild-type animals degenerate in the presence of β-amyloid. In contrast, cultures prepared from tau-/- animals show no signs of degeneration [218]. Together, these studies provide evidence that the presence of tau increases the susceptibility of neurons to β-amyloid and excitotoxic insults and suggest that tau is a downstream mediator of β-amyloid-induced toxicity (for review, see [220]). Indeed, β-amyloid influences the formation of NFT in tau transgenic mice [221-224]. For example, the clearance of β-amyloid by immunotherapy results in the removal of early-stage tau pathology in triple transgenic mice (3xTg-Ad) that normally develop β-amyloid plaques and NFT [224]. Conversely, when Lewis et al., (2001) crossed JNPL3 transgenic mice expressing P301L htau with Tg2576 transgenic mice expressing mutant APP, they found that the double mutants exhibited enhanced NFT pathology in the limbic system and olfactory cortex compared to JNPL3 mice [221]. Likewise, NFT tangle formation was aggravated when APP mutant mice (APP23 mice) were crossed with P301L tau transgenic mice, or when brain extracts from aged APP23 mice with β-amyloid deposits were intracerebrally infused in young P301L tau mice [223]. Gotz et al., (2001) reported that the injection of β-amyloid Aβ_42 _fibrils into the brains of P301L mutant tau transgenic mice markedly increased tau phosphorylation at S212/T214 and S422, as well as the number of NFT, along with neuropil threads and degenerating neurites in the amygdala of P301L, but not wild-type, mice [222]. Similarly, treating primary neuronal cultures [225-229] or neuronal-like cells lines [230,231] with fibrillar β-amyloid induces tau phosphorylation and toxicity. In primary hippocampal or cortical neurons, tau phosphorylation induced by treating cells with fibrillar β-amyloid is an early event followed by the somatodendritic accumulation of hyperphosphorylated tau in a soluble form that is not associated with microtubules and is incapable of binding microtubules *in vitro *[225]. Of interest, treatments that offer protection against β-amyloid-induced toxicity, like lithium [228] or the glutamate receptor antagonist, memantine [229], reduce tau phosphorylation. Together, these results suggest that β-amyloid triggers tau hyperphosphorylation, NFT formation and neurodegeneration.

## Concluding remarks

Because of the complexity of tau biology, it is expected that tau dysfunction contributes to toxicity via multiple mechanisms and at different stages of disease. The early axonal transport defects and synaptic damage could result from tau hyperphosphorylation and cytosolic accumulation whereas NFT, which may initially be formed as a protective mechanism to sequester toxic tau moieties, could eventually contribute to neuronal death. Unfortunately, despite the growing body of evidence in strong support for the involvement of pathologically modified tau and tau aggregates in neurodegeneration, the exact neurotoxic tau species have not been definitively identified. Both toxic gains of function and the loss of normal tau functions are believed to play a role in inducing neuronal death but the mechanisms by which this occurs remain elusive. Deciphering the causes and effects of tau-mediated toxicity are complicated by the various tau isoforms, the numerous abnormal tau modifications, as well as the likelihood that tau intermediates contribute to the progression of neuronal death at different phases of a lethal cascade of events. This may well explain why several lines of investigation have suggested diverse, and sometimes conflicting, mechanisms of tau toxicity. Some of the inconsistencies may reflect differences among tau isoforms, mutations and expression levels in the experimental models employed to examine tau-mediated neurodegeneration. Additionally, while these models have proven critical in our current understanding of tauopathies, it should be kept in mind that, in trying to recapitulate the formation of NFT within neurons by artificially overexpressing tau, certain subtle (but no less significant) changes in tau that contribute to the initiation and evolution of disease may be overlooked. Also complicating matters is the lack of knowledge regarding the functions carried out by tau beyond its well-established involvement in regulating microtubule assembly and stability. Tau associates with the plasma membrane and interacts with a number of proteins involved in cell signaling. Until these additional tau functions are better understood, the harmful consequences of aberrant tau modifications, and how they adversely influence these functions, cannot to be fully appreciated. Thus, continuing efforts should be made to further identify and characterize tau functions and how they are negatively affected by the accumulation of cytosolic tau, the altered cellular distribution of tau, abnormal tau modifications and changes in the balance of tau isoforms. Such investigations will not only offer insight on the mechanisms by which tau causes neuronal dysfunction and death, but may also help decipher the chronology of events involved in tau-mediated toxicity. Indeed, a better understanding of the initial events in tau-induced neurodegeneration is likely to provide the basis for early therapeutic strategies.

## Appendix 1: Key observations

- Tau plays a key role in the organization and integrity of the neuronal cytoskeleton by regulating microtubule dynamics. Hyperphosphorylated tau is the principal component of neurofibrillary tangles in AD and related tauopathies. The formation of NFT correlates with the severity of cognitive impairment in AD, suggesting that altered tau regulation plays an important role in the progression of tauopathies.

- Over 34 different tau mutations have been identified in cases of FTDP-17, indicating that tau abnormalities are sufficient to trigger neuronal death and dementia. Some of the identified tau mutations disrupt tau-microtubule interactions, accelerate filament formation or alter the ratio of 4R- to 3R-tau isoforms.

- The aggregation of tau into NFT, as well as pathological tau modifications (e.g. hyperphosphorylation), have been linked to tau-mediated neuronal death in experimental models of tauopathy. Cell culture and animal models in which wild-type or mutant tau is overexpressed often recapitulate key events observed in the progression of tauopathies, such as tau hyperphosphorylation and redistribution from axons to the somatodendritic compartment, synaptic damage, axonal degeneration, NFT formation and cell death.

- Many therapeutic strategies for AD focus on the pathogenicity of amyloid-β peptides. However, studies such as the one showing that decreasing tau levels ameliorates the amyloid-β-induced deficits in a mouse model of AD [217], provide evidence to warrant tau-directed therapeutic interventions.

## Appendix 2: Critical next steps

- ***What are the toxic tau species and how do they influence tau function? ***Evidence strongly supports the involvement of pathologically modified tau and tau aggregates in neurodegeneration but the exact neurotoxic species remain unclear. Tau dysfunction likely contributes to cellular demise via toxic gains of function as well as from the loss of normal tau function. To appreciate the detrimental consequences of a loss of tau function, more insight on all functions of tau, and how they are regulated by different tau isoforms or modifications, is critical.

- ***What is the sequence of events in tau-mediated death? ***Tau dysfunction likely contributes to cellular demise via multiple mechanisms and at different stages of disease. A better understanding of the causes of tau dysfunction (e.g. altered kinase/phosphatase activity, diminished tau clearance) may shed light on the initiating factors of tau pathology and provide insight on the first toxic tau intermediates. This information will be especially useful for the design of therapeutic strategies aimed at targeting the initial stages of tau-induced neurodegeneration.

- ***What tau-based therapeutic approaches will enhance the clinical outcome of patients with tauopathies? ***Even though many questions remain regarding tau's involvement in neurodegeneration, our current understanding can guide the development of tau-directed therapeutics. For example, knowledge that the accumulation of hyperphosphorylated tau plays a role in neurotoxicity, perhaps because PHF tau can no longer stabilize microtubules, inspired research on approaches aimed at inhibiting tau phosphorylation [232,233], eliminating pathological tau [83,234] or restoring microtubule function through the use of microtubule-stabilizing agents, like taxol [235]. These *in vivo *studies provide evidence that targeting events in the tau-cascade of neurotoxicity can be therapeutically beneficial. Thus, future efforts must include the development and testing of tau-based therapies.

## Abbreviations

AD: Alzheimer's disease; APP: amyloid precursor protein; CaMKII: calcium- and calmodulin-dependent protein kinase II; Cdk5: cyclin-dependent kinase-5; FTDP-17: frontal temporal dementia with Parkinsonism linked to chromosome 17; GSK-3: glycogen synthase kinase-3; htau: human tau; MARK: microtubule affinity regulating kinase; NF-M: neurofilament medium; NFT: neurofibrillary tangles; PDPK: proline-directed protein kinases; PHF: paired helical filaments; Pin1: protein interacting with NIMA 1; PKA: cyclic AMP-dependent protein kinase; PP1: protein phosphatase 1; PP2A: protein phosphatase 2A; SH3: Src homology 3 domains (SH3)

## Competing interests

The authors declare that they have no competing interests.

## Authors' contributions

TG was responsible for the writing of the review. LP and TG participated in the design and revision of the review. Both authors read and approved the final manuscript.
